# To what extent do Australian child and youth health, and education wellbeing policies, address the social determinants of health and health equity?: a policy analysis study

**DOI:** 10.1186/s12889-022-14784-4

**Published:** 2022-12-07

**Authors:** Clare Littleton, Caitlin Reader

**Affiliations:** Torrens University Australia, 81-88 Wakefield St, Adelaide, SA 5000 Australia

**Keywords:** Child, Youth, Family, Health policy, Education wellbeing policy, Social determinants of health, Health equity, Housing

## Abstract

**Background:**

Children and youth are an important population group requiring specific policies to address their needs. In Australia, most children and youth are doing well, however, certain equity groups are not. To address child and youth health equity in policy, applying a social determinants of health approach is considered best practice. For over 10 years governments in Australia have been called upon to address the social determinants of health, however, there has been limited action. Health and education departments are typically most involved in policy development for children and youth. To date, there have been limited systematic analyses of Australian child and youth health policies, and selected education wellbeing policies, with a social determinants of health and health equity focus and this study aims to contribute to addressing this gap.

**Methods:**

Policy analysis was conducted across 26 Australian child and youth health policies, and selected education wellbeing policies. We used an existing prior coding framework to understand the extent the social determinants of health and health equity were addressed. All policies were strategic level and only included if dated 2009 onwards.

**Results:**

Across 26 selected policies only 10% of strategies addressed the social determinants of health, demonstrating a lack of policy action. However, there is relatively even focus on all developmental stages, and an increased focus on youth. Equity is acknowledged across most policies with some groups receiving more attention including Aboriginal and Torres Strait Islander children. The social determinants of health addressed, to some degree, include early childhood development, education, parental workplace conditions, healthy settings, and housing, those least mentioned include public transport and regulation.

**Conclusion:**

This study demonstrates a lack of policy action on the social determinants of health within Australian child and youth health policy, and selected education wellbeing policies. Rather, the application of a siloed, and predominantly acute care approach. However, there is recognition of equity across all policies; an emphasis on housing as a determinant of health; and a link between health and education departments through education wellbeing policies, specifically addressing the issue of mental health.

## Background

Many children and youth living in Australia experience good health and wellbeing, however, recent reports suggest the government is not addressing the needs of the most vulnerable groups [1–4]. Compared to other Organization for Economic Cooperation and Development (OECD) countries Australia performs moderately in relation to child and youth health and wellbeing indicators [4]. However, there are serious concerns in some areas including mental health, discrimination, bullying, food insecurity, low and declining immunisation rates, and the cost of childcare [3]. A report by the Australian Child Rights Taskforce suggests equity groups of most concern are Aboriginal and Torres Strait Islander children, Lesbian, Gay, Bisexual, Trans, Intersex and Queer (LGBTIQ+) children, asylum seeker and refugee children, children living with a disability, and children from culturally and linguistically diverse backgrounds [4].

When developing health policy for children and youth aiming to reduce health inequities, a social determinants of health (SDH) approach is considered best practice [5, 6]. In this study we define a SDH approach (or lens) in policy as strategies that ‘improve the conditions under which people live: including secure, safe, adequate, and sustainable livelihoods, lifestyles, and environments, including housing, education, nutrition, information exchange, child care, transportation, and necessary community and personal social and health services’ ([7]; p.622). The recently developed a ‘health in all policies’ framework exemplifies the above approach, offering a way for governments to address health and wellbeing across sectors [8]. Thus, there is a need to consider a broad approach to health, including prevention, through a SDH approach, across cross departments [9–11].

Table 1 (below) describes the social determinants of health particularly important for children, youth and families [3, 5, 10, 12]:

**Table 1:** Social determinants of child and youth health particularly important for children, youth and families

Main category	Sub-category
Early childhood development (ECD)	Physical/social/emotional, and language/cognitive domains of development
Education	Primary, secondary and tertiary education
Social and economic conditions	Affordable housing, parental educational levels, social connectedness, positive family relationships, income/employment, adequate/affordable childcare and flexibility in the workplace for parents, and access to clean water and healthy food
Sociocultural conditions	Colonialism, class, gender, racism and other forms of discrimination
Healthy settings	Safe local communities, urban planning, green space, childcare, schools, workplaces
Access to health services	Affordability, availability, acceptability
Corporate, environmental, and global forces	Food production and the marketing of global corporations, media consumption and regulation, pollution/climate change, and materialism

It is also important to note a SDH approach is compatible with a range of the UN sustainable development goals including Goal 3 Good Health and Well-being which aims to ‘ensure healthy lives and promote well-being for all at all ages’ [13]. In addition, for children and youth, education and health outcomes are intrinsically linked, especially in relation to child development, with the effects continuing across the life course [9]. A recent Lancet article suggests ‘education shapes lives’ and provides a way to lift people ‘out of poverty’ [14; p.e361]. Furthermore, through the global COVID-19 pandemic education has been highlighted as vital to the overall health and wellbeing of children and families across the globe [14]. Therefore, the SDG 4 Quality Education which aims to ‘ensure inclusive and equitable quality education and promote lifelong learning opportunities for all’, is one of the most important cross department considerations for those developing policy for this population group [13]. Other scholars suggest there is a ‘natural link’ between health and education policy for children stating ‘health and education sectors have long been seen as natural partners with mutually beneficial goals’ ([15]; p.1). Yet, many health and education departments develop policy in a siloed manner claiming to have different priority areas and not understanding each other’s language [16].

In recent times there has been much attention paid to translating evidence on SDH into policy to reduce health inequities in Australia [17, 18]. However, there has been limited policy action in this area [5]. In 2013, we conducted a study on all Australian child and youth health policy using the same prior coding framework used here. We found only 10% of strategies addressed the SDH. The SDH addressed most included early childhood development, education, and healthy settings [5].

After a 10-year gap, we conducted a similar systematic analysis of current Australian child and youth health policy with a SDH and health equity focus. In addition to the 2013 (published 2016) study we also included selected education department wellbeing policies.

When we conducted our last study in 2013 (published 2016), there was some momentum for funding of health policies addressing prevention, specifically through the National Partnership Agreement on Preventative Health NPAPH [19]. This was due to the potential cost of chronic disease to the health system and a range of advocacy efforts to promote a SDH approach [6]. In addition, the government elect at this time appeared to be more favourable to the social aspects embedded with a social determinants approach, as demonstrated with the emphasis on early childhood development policy at state and federal level policy [5, 6]. However, in 2013 a new conservative government was elected in Australia. Subsequently, in the 2014 health budget there was a decrease in focus and funding in this area, including the cessation of the NPAPH [19]. This has been attributed to a neo-liberal style government placing an emphasis on economics over social priorities [18]. Therefore, over the 10 years since our last study, the political landscape has changed in Australia, and there appears to be less political will to take action on the SDH. This pattern is similar in other high-income countries, depending on the government elect at the time [20].

Therefore, to better understand how the health policy environment has changed over the past 10 years, and influenced the adoption of a SDH approach in Australia, with a specific focus on children and youth, in this study we address the question:

*To what extent do Australian child and youth health policy and selected education wellbeing policies address the social determinants of health and health equity?*

## Methods

An existing prior coding framework was adapted to analyse a selected set of 26 child and youth health, and selected education wellbeing policies. This framework was originally developed by Fisher et al. (2015) [21] to analyse strategic level National and state level health policy in Australia, and adapted by Phillips et al. (2016) [5] to be more specific to child and youth health policy. This method enables the researcher to conduct a systematic review of child and youth policies with a focus on the extent the SDH and health equity are addressed. The framework draws on seminal scholars from this area of scholarship including Baum [18], Dahlgren and Whitehead [22], and Carter [23] and allows the researcher to understand the status quo, or extent a set of policies addresses the SDH and health equity, at a particular point in time. The intention of the Fisher (2015) [21] framework is to provide ‘an effective way to interrogate health policies on key points raised in recent literature about the translation of evidence on SDH into policy’ (p.1). As suggested by the authors, this framework focusses on ‘analysis of the content of health policy documents, and does not consider issues of policy implementation’ ([21]; p.2). Therefore, one of the limitations of this study is it is not possible to code whether strategies are short, medium, or long term, especially in relation to implementation.

The process of analysis begins with a pre-determined set of categories (see Table 2) used to code chunks of text from each policy. In doing so it is possible to quantify and categorise the focus of strategies and objectives, provide detailed quotes or examples, and therefore better understand the focus of policies as related to the literature on the social determinants of child and youth health and health equity. More details about these methods will be provided below.

**Table 2:** Coding structure

Main Categories (codes)	Codes (sections of text can be coded against several codes)
(a) Goals	5 codes re gains in intended gains in health status specified as either: • Average health • Subject to ill health • Equity groups • Close the Gap • Across the gradient
(b) Recognition of evidence on the SDH & HE	• Acknowledge • Audit
(c) and (d) Objectives & Strategies *Both of the above categories are coded against the same codes.*	• Environmental health • Research • Policy development and governance • Workforce • Health service quality • Health service access • Collaboration between health services • Health promotion and disease prevention (Individual behavioural) • Community engagement • Cross sector activity • Social determinants of health (other than health service access) • Home environment • Early childhood development • Education • Health settings • Employment/workplace conditions • Housing • Urban planning • Public transport • Regulatory measures • and Reducing social inequities *Note: Strategies are all double coded against intended outcomes in regards to health equity*

### Document analysis

Document analysis is an appropriate method for this study because, as suggested by Patton [24], it allows the researcher to make sense of a range of documents using a systematic method to deconstruct the text and assess the status quo. Public health researchers study policy documents in order to better understand current policy decisions and promised action in relation to best practice evidence in a particular area. Other public health scholars have also used this method to focus on government policies and better understand how to improve equity and overall health outcomes for particular population or disease groups [5, 23, 25–27].

### Policy selection

Policies were selected for analysis following a review of all federal and state/territory strategic (as opposed to operational) health department, and selected education wellbeing policies dated from 2009 onwards. Policies with an end date prior to 2010 were not included ie 2000–2009, however, policies with a start from 2009 which extended beyond 2010 were included. This is because, due to the end date (ie 2009–2020), these policies were still considered up to date and in the scope of this study, and therefore, worthy of inclusion. All policies selected for analysis were publicly available through health and education government websites. To select the policies two researchers conducted a rapid review of all Australian health department and education websites, and applied a selection criterion (see below). At this stage we compiled a list of suitable policies and excluded any policies not matching our criteria. Health policies for this study were selected if the title of the policy addressed children, youth, paediatric, adolescent, youth health, or families. Education policies were selected if the title of the policy included ‘wellbeing’. We also selected whole of government policies addressing child and youth health issues and assigning responsibility to the health or education department, and, health promotion or disease prevention policies with a chapter or extensive section dedicated to children. There were several policies that did not explicitly record a date or date range. To ensure currency, the release date of the policy was crossed checked through a government media release. For any other policies where we were unsure of currency, we called the relevant Health or Education Department to confirm. At this stage no policies were excluded. This selection process and criteria resulted in the inclusion of 26 policies, as shown in Table 3, providing a set of strategic child and youth health and education wellbeing policies to analyse. This search occurred between October and December 2021.

**Table 3:** Policies selected

Selected policies	
**Strategic health service plans**	
1. Strong Families – Safe Kids Implementation Plan 2016–2020 (TAS)	
2. Aboriginal Family Health Strategy 2011–2016: Responding to Family Violence in Aboriginal Communities (NSW)	
3. Women’s, Children and Youth Health Plan 2021–2031 (draft – in consultation)	
4. WA Youth Health Policy 2018–2023 - Strong Body, Strong Minds, Stronger Youth	
5. Healthy Safe and Well: A strategic Health Plan for Children, Young people and Families 2014–2024 (NSW)	
6. Children’s Health and Wellbeing Services Plan 2018–2028 (QLD)	
7. Aboriginal and Torres Strait Islander Health and Wellbeing Services Plan 2018–2023 (QLD)	
**Comprehensive and cross sector approaches to child and youth health and well-being**	
8. The ACT Children and Young People’s Commitment 2015–2025	
9. Tasmanian Child and Youth Wellbeing Framework 2018	
10. NSW Youth Health Framework 2017–24	
11. It takes a Tasmanian Village: Child and Youth Wellbeing Strategy (2021)	
12. The Best Opportunities in Life: Northern Territory Child and Adolescent Health and Wellbeing Strategic Plan 2018–2028	
13. A great start for our children: Statewide plan for children and young people’s health services to 2026 (QLD)	
14. Child and youth framework Six Domains – Strategic Directions 2021–2024 (TAS)	
**Policies addressing specific childhood health issues**	
15. NSW Healthy Eating and Active Living Strategy: Preventing overweight and obesity in New South Wales 2013–2018	
16. Towards Zero Growth: Healthy Weight Action Plan 2013 (ACT) – Note: could also be categorised below	
**Broader health policies (with dedicated a chapter or section to children’s health)**	
17. Primary Prevention Plan 2011–2016 (section on children and youth) (SA)	
**National**	
18. National Framework for Child and Family Health Services – Secondary and Tertiary Services 2015	
19. National Children’s Mental Health and Wellbeing Strategy 2021	
20. National Framework for Protecting Australia’s Children 2009–2020	
21. National Action Plan for Health of Children and Young People 2020–2030	
22. National Framework for Health Services for Aboriginal and Torres Strait Islander Children and Families 2018	
**Education policies with health and/or well-being in title**	
23. Child and Student Wellbeing Strategy – safe, well, and positive learners 2018–2021 (TAS)	
24. The Wellbeing Framework for Schools (NSW) 2015	
25. Wellbeing for Learning and Life: A framework for building resilience and wellbeing in children and young people (SA) 2013	
26. Student Learning and Wellbeing Framework (QLD) 2018	

To better understand what has changed since the last analysis in 2013 (published in 2016), the following section further analyses and compares differences between the policies selected in 2013 and 2021.

a. Two policies remain the same:

- Aboriginal Family Health Strategy 2011–2016: Responding to Family Violence in Aboriginal Communities (NSW)
- Primary Prevention Plan 2011–2016 (section on children and youth), South Australia (SA)

b. Two policies have been updated with a more up to date policy with the same focus, and format:

- Youth Health Policy 2011–2016: Healthy bodies, healthy minds, vibrant futures, New South Wales (NSW) updated to *WA Youth Health Policy 2018–2023 - Strong Body, Strong Minds, Stronger Youth*
- NSW Governments Plan for Preventing Overweight & Obesity in Children, Young people & their Families 2009–2011, New South Wales (NSW) updated to *NSW Healthy Eating and Active Living Strategy: Preventing overweight and obesity in New South Wales 2013–2018*

c. Six policies were not included, as did not fit into the 2021 section criteria (see reason below):

- Supporting families early-SAFE START strategic policy 2009, New South Wales (NSW) - *end date prior to 2010*
- Supporting Families Early Package—maternal and child health primary health care policy 2009, New South Wales (NSW) - *end date prior to 2010*
- Strategic framework for paediatric health services in Victoria 2009, Victoria (VIC) - *end date prior to 2010*
- CAMHS (Child and Adolescent Mental Health Services) in communities 2006, Victoria (VIC) - *end date prior to 2010*
- Victorian (VIC) Public Health and Well Being Plan 2011–2015 (chapter early childhood and education) *- chapter on children removed in updated policy*
- Preventative Health—Strategic Direction 2010–2013 (section on children), Queensland (QLD) *- chapter on children removed in updated policy*

d. Seven policies were replaced with a relatively new focus and format:

- Child Health & Parenting Service (CHAPS)-Strategic Plan 2009–2014, Tasmania (TAS) *replaced by Strong Families – Safe Kids Implementation Plan 2016–2020 (TAS)*
- Children, Youth and Women’s Health Service (CYWHS) Strategic Plan 2011–2015, South Australia (SA) *replaced by Women’s, Children and Youth Health Plan 2021–2031 SA (draft – in consultation)*
- Our children our future: A framework for Child and Youth Health Services in Western Australia 2008–2012, Western Australia (WA) *replaced by WA Youth Health Policy 2018–2023 - Strong Body, Strong Minds, Stronger Youth*
- Our Children Our Future 2011–2021, Tasmania (TAS) *replaced by Child and Youth Wellbeing Strategy: It takes a Tasmanian Village (TAS)*
- ACT Children’s Plan: Vision and building blocks for a child-friendly city 2010–2014, Australian Capital Territory (ACT) *replaced by The ACT Children and Young People’s Commitment 2015–2025*
- Guidelines on the Management of Sexual Health Issues in Children and Young People 2011, Northern Territory (NT) *replaced by The Best Opportunities in Life: Northern Territory Child and Adolescent Health and Wellbeing Strategic Plan 2018–2028*
- Keep Them Safe: A shared approach to child well being 2009–2014, New South Wales (NSW) *replaced by Health Safe and Well: A strategic Health Plan for Children, Young people and Families 2014–2024 (NSW)*

### Coding

The analysis of policies was conducted using the above coding structure (Table 2) by one researcher between November 2021 and March 2022. Over half the analysis was then cross checked by another member of the research team. Comments and reflections were recorded throughout analysis and, at this stage, if there were any issues or disagreements they were discussed and resolved. More details about the coding procedure can be found in Fisher et al. (2015) [21] and Phillips et al. (2016) [5].

## Results

### Targeted age group

A range of ages were covered from 0 to 24 years. Overall, 13 policies focussed on children and youth (0–24 years); 4 policies focussed on children (0–18 years); 2 policies focussed on young people only; 2 policies focussed on Aboriginal children and youth; and 1 policy focussed on Aboriginal families and communities. The four selected education policies were focussed on school aged children (5–18 years). Research suggests the different stages of childhood are early childhood (0–8 years), middle childhood (9–12 years), young people or youth (12–24 years) [2, 7]. Compared to our 2013 study (published in 2016), we found an increased awareness in policy about the need to address different developmental stages. Across all health and education policies selected for this study strategies were relatively evenly spread across early childhood (0–8 years), middle childhood (8–12 years), and young people (12–24 years). More details of the selected policies are provided in Table 3.

### Goals and equity groups

Most of the goals identified (21) were focussed on the overall health and wellbeing of all children, young people, and families. For example, *‘to improve the health and wellbeing of young Territorians, aged from birth to 24 years’* (The Best Opportunities in Life: Northern Territory Child and Adolescent Health and Wellbeing Strategic Plan 2018–2028); *‘To optimise the health and wellbeing of young people in WA’* (WA Youth Health Policy 2018–2023 - Strong Body, Strong Minds, Stronger Youth); *‘support the learning and life opportunities of all children and young people, and seek to help make them strong, creative and resilient learners, to set the trajectory for lifelong wellbeing’* (Wellbeing for Learning and Life: A framework for building resilience and wellbeing in children and young people (SA) 2013). There were 11 goals where equity groups, and/or vulnerable or at-risk children were specifically mentioned. The equity group mentioned most were Aboriginal and Torres Strait Islander children, for example, *‘The goal of the Strategy is to ensure that all Aboriginal people in NSW live safe and healthy lives free of family violence’* (Aboriginal Family Health Strategy 2011–2016: Responding to Family Violence in Aboriginal Communities NSW); *‘The principles described in this framework aim to redress the inequity Aboriginal children and young people experience in child protection, health and education’* (Wellbeing for Learning and Life: A framework for building resilience and wellbeing in children and young people SA 2013)*.* Those equity groups mentioned, but less often, were Culturally and Linguistically Diverse (CALD), refugee, out of home care, LGBTQI+ children; or those children living with a disability or complex health conditions, in poverty, experiencing homelessness or domestic and family violence, and in rural, regional and remote areas. Four goals specifically mentioned a commitment to closing the gap, for example, *‘build on the Queensland State Governments commitment to ‘Closing the Gap’*. The Closing the Gap Campaign was launched in 2008 by the Australian Government with the aim of closing the health and life expectancy gap between Aboriginal and Torres Strait Islander peoples and non-Indigenous Australians within a generation [28].

### Reaching full potential and giving children a say

One of the ways policies referred to positive health outcomes was to make statements about children reaching their full potential (8). This was particularly evident when policies suggested children should be given a ‘voice’ and be part of decision making. For example:

*‘All children and young people (0–25 years) across the ACT will have the opportunity to reach their potential, make a contribution, participate in decision making and share the benefits of our community’* (The ACT Children and Young People’s Commitment 2015–2025).

*‘This includes the need for all involved to actively listen to, and respect, children’s voices in their own care decisions’* (National Children’s Mental Health and Wellbeing Strategy 2021).

*‘The DEC commitment to wellbeing is for our schools to support students to connect, succeed and thrive at each stage of their development and learning; to provide opportunities that are age rigorous, meaningful and dignified; and to do this in the context of individual and shared responsibility underpinned by productive relationships that support students to learn’* (The Wellbeing Framework for Schools (NSW) 2015).

This was backed up by almost half the policies stating that during the development of the policy they had consulted with either children or youth and/or parents, or key stakeholders (experts in this area) from outside government.

Notably, the policies that incorporated ‘children’s voices’ into decision making were the more recently written health policies and education wellbeing policies, with a focus on child rights and the SDH.

### Acknowledgement and auditing of evidence on the SDH

As outlined in Phillips et al. 2016 [5], as part of the coding structure we identified acknowledgement of evidence on the SDH when a simple statement about the evidence was made, and auditing of evidence, when specific evidence was described and/or explained in more depth [5, 21, 23].

All policies in this study acknowledged the SDH and health equity to some degree, with the extent they did so varying. Across 26 policies there were 110 references coded as acknowledging the SDH and health equity, and 67 as auditing the SDH and health equity.

The below quotes demonstrate how selected policies acknowledged evidence on the SDH.

*‘The wellbeing of children is interwoven with their parents, families and caregivers who need the right kind of support to provide a nurturing environment for their children’* (It takes a Tasmanian Village: Child and Youth Wellbeing Strategy (2021).

*‘Actions to influence healthy eating and physical activity require a comprehensive approach. This recognises the interaction of individual, societal and environmental factors that impact directly and indirectly upon behaviours that have led to weight gain over the last fifteen years across NSW’* (NSW Healthy Eating and Active Living Strategy: Preventing overweight and obesity in New South Wales 2013–2018).

Interestingly, the National Children’s Mental Health and Wellbeing Strategy 2021 acknowledges the evidence on the social determinants of mental health in a section titled *‘community driven approaches’* but also makes a clear statement about the department’s willingness to translate this evidence into health policy: *‘While addressing all social determinants of child mental health and wellbeing is beyond the scope of this strategy, they should remain key considerations as part of community-driven approaches’.* Recent research suggests this version of addressing the SDH in Australian policy is driven by policy actors adopting a diversion tactic to fit a SDH approach into the neo-liberal individual behavioural discourse within government [6, 16].

The below quotes demonstrate how selected policies audit evidence on the SDH.

*‘Healthy homes provide shelter, privacy, safety and security, support health and education and significantly impact workforce participation. Many, particularly Aboriginal children, young people, and their families live in housing that is unsatisfactory. Factors such as inadequate water and sewage systems, waste collection, electricity and housing infrastructure contribute to this unacceptable living conditions’.* (The Best Opportunities in Life: Northern Territory Child and Adolescent Health and Wellbeing Strategic Plan 2018–2028).

*‘access to health care is complicated by psychosocial factors which include inadequate access to financial support, lack of or disengagement from education or employment, lack of safe or adequate housing, mistrust of health services, stigma related to mental health issues, (and) language barriers’* (WA Youth Health Policy 2018–2023 - Strong Body, Strong Minds, Stronger Youth).

### Objectives

In this study we define objectives as text ‘describing an operational goal (that is, an intended outcome) for improved system or service performance in an area of departmental activity’ ([21]; p. 9).

Across 26 policies we identified 428 objectives, nearly half (196) were health services related, followed by SDH (115), health promotion and prevention (individual) (49), policy development and governance (36), and cross sector activity (32). The numbers here refer to the number of objectives, drawn from across policies, coded under each category. Table 4 illustrates some of the phrases coded under each category.

**Table 4:** Coded under objectives

Code	Phrase from policy
Health Services	*‘Drive equitable, effective and coordinated health services that optimise the health and wellbeing of young people in WA’* (WA Youth Health Policy 2018–2023 - Strong Body, Strong Minds, Stronger Youth); *‘Improved health and wellbeing system to support our people’* (Child and youth framework Six Domains – Strategic Directions 2021–2024 TAS)
Social Determinants of Health	*‘Support parents and carers during the first 1000 days’ (*It takes a Tasmanian Village: Child and Youth Wellbeing Strategy 2021); *‘Support DECS to improve literacy and educational outcomes of children from low socioeconomic status and other vulnerable backgrounds’* (Primary Prevention Plan 2011–2016 SA)
Health Promotion and Prevention (Individual)	*‘Promote and refine preventive health strategies and interventions addressing sleep, nutrition, physical activity, and overweight and obesity’* (National Action Plan for Health of Children and Young People 2020–2030)
Policy Development and Governance	*‘(Ensure) strong governance and role clarity exists for services at all levels of the system’* (A great start for our children: Statewide plan for children and young people’s health services to 2026 QLD)
Cross Sector Activity	*‘Build strong and safe communities that promote, protect and create awareness of children and young people’s rights through a shared understanding across government, community and media’.* (The ACT Children and Young People’s Commitment 2015–2025)

### Strategies

In this study we describe strategies as text ‘describing intended actions or kinds of action within an area of departmental activity’ ([21]; p.9).

We found a total of 1359 strategies, approximately two thirds were related to health services delivery (904), including 49 strategies focussed on acute care health services access, followed by cross sector activity (234), policy development and governance (209), the SDH (143), and health promotion and prevention (individual) (74). Table 5 illustrates some of the phrases coded under each category.

**Table 5:** Phrases coded under strategies

Code	Phrase from policy
Health Services	*‘Trialling sites with innovative service delivery models that integrate face-to-face and telehealth consultations, digital interventions, and phone helplines’* (National Children’s Mental Health and Wellbeing Strategy 2021); *‘Increase the use of the clinical prioritisation criteria when referring into the Queensland public hospital system’ (*A great start for our children: Statewide plan for children and young people’s health services to 2026 (QLD); *‘Expansion and improved access to mental health services in urban, regional and remote areas’* (The Best Opportunities in Life: Northern Territory Child and Adolescent Health and Wellbeing Strategic Plan 2018–2028)
Cross Sector Activity	*‘NSW Health will strengthen relationships with other health services and cross sector partners to provide integrated and coordinated care’.* (NSW Youth Health Framework 2017–24)
Policy Development and Governance	*‘Develop and implement an ACT Government school food and drink policy with supporting guidelines that will mandate the implementation of the National Healthy School Canteen Guidelines in all ACT schools’* (Towards Zero Growth: Healthy Weight Action Plan 2013)
Social Determinants of Health	*‘Promote safe inclusive community environments that allow children and young people to play, explore, grow and have experiences that promote positive development’* (The ACT Children and Young People’s Commitment 2015–2025); *‘Restrict the advertising of unhealthy foods within the government’s regulatory control … there is a particular need to address marketing directed at children in close proximity to schools, playgrounds and child care centres’* (Towards Zero Growth: Healthy Weight Action Plan 2013*); ‘Develop approaches to increase access to and provision of fresh fruit and vegetables to remote communities and among populations experiencing disadvantage’* (National Action Plan for Health of Children and Young People 2020–2030); *‘(provide) free sanitary items for all government schools for students in need’* (It takes a Tasmanian Village: Child and Youth Wellbeing Strategy (2021). *‘Promote evidence-based initiatives through the trial of the Housing for Health program’* (Aboriginal and Torres Strait Islander Health and Wellbeing Services Plan 2018–2023 QLD)
Health Promotion and Prevention (Individual)	*‘Implement community based healthy lifestyle interventions in disadvantaged communities including addressing alcohol abuse’* (National Framework for Protecting Australia’s Children 2009–2020); *‘Improve front-of-pack labelling and support interpretation of label changes with targeted social marketing campaigns’* (NSW Healthy Eating and Active Living Strategy: Preventing overweight and obesity in New South Wales 2013–2018); *‘Distribute targeted promotion and prevention resources for Aboriginal and Torres Strait Islander families and young people’* (ATSI Health and wellbeing services plan Aboriginal and Torres Strait Islander Health and Wellbeing Services Plan 2018–2023 QLD)

### SDH

In relation to the SDH, the focus of this study, only 10% of strategies were coded in this category. This demonstrates a lack of action on the SDH and the continuation of an unbalanced health system in Australia, predominantly focussing on an acute care approach rather than addressing the SDH known to reduce health inequities. To better understand the SDH that were present in the selected set of policies, as per our 2013 study, we have divided the SDH into a range of codes (see Table 1). As a result, we can see early childhood development (ECD), education, parental workplace conditions, and healthy settings have remained a focus. Also, urban planning, and in particular housing, has received slightly more attention, with a specific focus on youth. Finally, to a limited degree there has been a focus on public transport and regulatory measures. See below a more detailed explanation for each code.

### Early childhood development (ECD)

We found 27 strategies addressing ECD. Action on ECD placed an emphasis on promoting inclusive community services or environments to support families in the first 1000 days of a child’s life. For example, *‘The Child Health and Parenting Services Sustained Nurse Home Visiting Program will provide targeted home visits to families who are identified as having complex needs and would benefit from additional child health support in the first 1,000 days’...’this will include free access to speech pathologists, psychologists and social workers for every child and family attending a Child and Family Learning Centre’* (It takes a Tasmanian Village: Child and Youth Wellbeing Strategy (2021)*; ‘continue to build the capacity of families and communities to provide responsive and appropriate care that promotes children and young people’s optimum development through the provision of culturally inclusive universal human services across sectors and systems’* (The ACT Children and Young People’s Commitment 2015–2025).

There was also an emphasis on parents being given the resources to understand the importance of ECD. For example: *‘The Basics’ deploys knowledge about effective care giving in the first few years of their child’s life’ (*It takes a Tasmanian Village: Child and Youth Wellbeing Strategy (2021); *‘The Healthy Beginnings telephone-based support service to promote healthy eating and physical activity to parents of children 0-2 years’* (NSW Healthy Eating and Active Living Strategy: Preventing overweight and obesity in New South Wales 2013–2018). The other focus was positive development through the provision of inclusive and accessible early childhood education for parents. For example, *‘Support education programs that train parents and caregivers to increase infant attachment and security’* (National Action Plan for Health of Children and Young People 2020–2030); *‘Routinely offer evidence-based parenting programs to parents and carers at key developmental milestones for their child’* (National Children’s Mental Health and Wellbeing Strategy 2021).

### Education

We found 13 strategies related to education. The main focus was to provide a quality education throughout childhood, for example: *‘Implement a world class ACT curriculum from preschool to year 12 in alignment with the Australian Curriculum’* (The ACT Children and Young People’s Commitment 2015–2025). Another focus area was alternate pathways for children who are disengaged in education, have learning difficulties, live remotely, or are disadvantaged because of child protection issues. For example: *‘Proactive outreach procedures should be developed to respond to student disengagement, using trauma informed approaches’* (National Children’s Mental Health and Wellbeing Strategy 2021); *‘support children and young people to remain engaged or re-engage in education’* (Safe Kids Implementation Plan 2016–2020 TAS); *‘Aboriginal children and young people will have an individual personalised learning pathway’* (The Wellbeing Framework for Schools 2015 NSW); *‘continue to support principals, assistant teachers and educators to deliver a quality preschool program to children in small, remote and very remote preschools’* (The Best Opportunities in Life: Northern Territory Child and Adolescent Health and Wellbeing Strategic Plan 2018–2028). An important development in cross department strategies between health and education departments is in the area of mental health. For example, in the National Children’s Mental Health and Wellbeing Strategy 2021, one of the four main sections is dedicated to early childhood and primary school settings: ‘*the strategy emphasises the important role that educational settings play in promoting mental health and wellbeing in children, and discusses the additional supports that may be required for educators to continue to build positive wellbeing cultures’* (National Children’s Mental Health and Wellbeing Strategy 2021). The role of schools is also acknowledged in the SA Primary Prevention Plan *‘Schools play a vital role in building a positive environment and creating a sense of belonging. They build knowledge, protective skills and positive attitudes in relation to important factors such as parental and peer relationships, conflict resolution, bullying prevention, social and family norms, resilience, communication skills, goal-setting, strong cultural/ ethnic identity, health information and help-seeking behaviour’* (Primary Prevention Plan 2011–2016 SA).

### Parent workplace conditions/healthy settings

There were 12 strategies related to parent workplace conditions, but they overlapped with the healthy settings (35) code where there was a fairly narrow view of healthy settings, with workplaces being encouraged to ensure improved choices for healthy food, beverages and exercise. For example*: ‘Implement a Chief Minister’s Award scheme that rewards healthy workplaces and food outlets’* (Towards Zero Growth: Healthy Weight Action Plan 2013). There were also strategies where parents at work were encouraged to engage in mental health training, for example: *‘target parents in workplace mental health programs, with a particular focus on new parents during the return to work phase’* (National Children’s Mental Health and Wellbeing Strategy 2021). In addition, some healthy settings strategies focussed on addressing marketing aimed at children, for example: *‘address marketing directed at children in close proximity to schools, playgrounds and child care centres’* (Towards Zero Growth: Healthy Weight Action Plan 2013). There was also a focus on healthy settings encouraging children to be physically active, for example: ‘*access to recreational and natural environment setting to encourage physical activity’* (Tasmanian Child and Youth Wellbeing Framework 2018*).* There were several strategies extending the healthy settings code to include support for wellbeing, including sanitary pads in workplaces and schools, for example: *‘ensure that no female student in Tasmanian government schools will be absent because they are unable to access sanitary products’* (It takes a Tasmanian Village: Child and Youth Wellbeing Strategy (2021). This strategy may be due to recent advocacy work across Australia on menstruation through the South Australian Commissioner for Children and Young People who released a report in 2021 titled ‘Menstruation Matters: The impact of menstruation on wellbeing, participation and school attendance’ [29].

### Urban planning and housing

Urban planning strategies (14) were mostly related to creating more walkable and cycle friendly spaces in cities, for example *‘designing urban centres and housing to support physical activity and active transport’* (NSW Healthy Eating and Active Living Strategy: Preventing overweight and obesity in New South Wales 2013–2018); *‘Create car parking and other incentives which encourage active travel (walk/cycle/ bus) and discourage private transport for entire journeys into town centres’* (Towards Zero Growth: Healthy Weight Action Plan 2013). Strategies related to housing occurred 16 times, with an emphasis on increased access and affordability for children, youth, and families, for example *‘increase access to housing that is affordable, safe and secure for children, young people and their families’* (The ACT Children and Young People’s Commitment 2015–2025). Across the selected set of policies there was a specific focus on housing for youth, most notably in the Northern Territory and Tasmanian policies, where the emphasis is on reducing homelessness.

For example, in the policy: It takes a Tasmanian Village: Child and Youth Wellbeing Strategy (2021), the strategies relating to housing include:

**Table:** 

• Provide stable housing and supports for homeless youth. • This will be achieved through: provision of family-like residential care for young people under 16 who are unable to live at home through the Lighthouse Project; Youth Wellbeing Officers who will provide community youth support and advice; housing for youth transitioning from statutory services or shelters including Youth Coaches to support these transitions; and identification of properties suitable for conversion to share housing for young people. • It will include a residential care pilot program for young people under 16 who are not in the care of the state and are unable to live at home. The pilot will run over 3 years and provide a family like environment, accommodation and a program of therapeutic care with a focus on family restoration. • It will provide 20 modular youth homes across four sites around the State. • This initiative will identify 10 Housing Tasmania properties suitable for conversion into three to four-bedroom share housing properties for young people.	

It is also clear from the community consultation during the development of the above policy, when children are consulted, they see housing as an important determinant of health: *‘I hope that builders can make more houses so that homeless people can have a home like us … ’ aged 9*; *‘Socialism, climate action so that young people have a world to live in, more mental health facilities, affordable housing, free education … ’ aged 21; ‘Nana to live in tassie again, so she needs a house … ’ aged 5.*

Note: the above strategies are expanded on considerably in Tasmania’s Affordable Housing Strategy 2015–2025.

In the policy, Best Opportunities in Life: Northern Territory Child and Adolescent Health and Wellbeing Strategic Plan 2018–2028 the strategies related to housing include:

**Table:** 

• increasing the number of remote public housing properties through HomeBuild NT. • increasing the number of bedrooms and living spaces in remote communities through Room to Breathe. • additional repairs and maintenance of remote community housing. • increased access to Government Employee Housing for Aboriginal government employees. • improved housing amenity across remote Aboriginal communities. • houses are maintained and tenants supported to meet their responsibilities, resulting in improved longevity of housing.	

Note: the above strategies are expanded on considerably in Northern Territory homelessness strategy 2018–2013.

Other strategies under the SDH include public transport (3) and regulatory measures (7).

## Discussion

The aim of this study was to understand the extent current Australian child and youth health policies, and selected education wellbeing policies, address equity, and propose action on the SDH. We developed a selection criteria for the inclusion of 26 suitable health and education wellbeing policies. We then applied an existing prior coding framework (Fisher et al. 2015) [21] allowing the systematic analysis of state level and federal policies, with a specific focus on the SDH and health equity.

Our findings highlight recent developments in the Australian health policy environment for children and youth. We also consider the importance of the link between health and education departments through the inclusion of selected education wellbeing policies.

Building on a similar selection criterion in 2013, there has been an increase in National level policies (5) focusing on children and youth in Australia. This may reflect the recent agenda from the Australian Human Rights Commission working on a co-ordinated approach in addressing the Convention on the Rights of the Child (CRC) through law, policy, and a Minister to represent children and youth, at a Federal level [30].

One of the key findings from this study, and improvement compared to the 2013 study, is the acknowledgement of child and youth voices, in the development of policy. This is likely due to the work of National and state level Children’s Commissioners/Guardians upholding Article 12 of the United Nations Rights on the Convention of the Child, specifically that ‘*Children have the right to have their views taken into account in matters that affect them*’ [31]. Others have also noted this improvement suggesting while there is some progress in governments listening to youth voices during policy development, there is still room for improvement [27].

The government in the Northern Territory has significantly improved policy outputs for children and youth with a comprehensive policy title ‘The Best Opportunities in Life: Northern Territory Child and Adolescent Health and Wellbeing Strategic Plan 2018–2028’*.* The above policy is one of the only policies in this study to use a SDH framework and make specific and measurable commitments in this, including naming budgetary values. There are also 6 policies using the ‘Nest Framework’, Australia’s first evidence-based framework for national child and youth wellbeing, focussing on six wellbeing domains [32]. The ‘Nest Framework’ has many of the SDH embedded within it. The adoption of this framework reflects the influence of the Australian Research Alliance for Children and Youth (ARACY), a National NGO which aims to ‘improve the lives and prospects of children and young people in Australia’ [33]. Interestingly, the ‘Nest Framework’ is used across both health and education policies in this study and appears to offer a way of breaking down barriers in cross department work between health and education. There is also evidence that when addressing mental health and resilience, Australian health departments consider the education department as an important partner. Through the above findings, and the inclusion of wellbeing policies within NSW, SA, TAS and QLD education departments, the Australian government appears to recognize education as a determinant of health. Furthermore, that cross department activity between health and education is progressing. However, more work is needed. The above discussion is also clearly linked to SDG 4 Quality Education which aims to ‘ensure inclusive and equitable quality education and promote lifelong learning opportunities for all’ [13].

Another key finding from this study is a more evenly distributed focus across early childhood (0–8 years), middle childhood (9–12 years), and young people or youth (12–24 years). In particular an increased focus on youth health, both through dedicated youth health policies, and embedded in broader health policies. Waller et al. [27] also found an increase in the number of dedicated youth health policies across Australia. This finding is contrary to our 2013 study where the focus was predominantly on early childhood (0–8). This is an important improvement because while focussing on early childhood is fundamental to health across the life course [9], when there is limited focus as children move through the older years of childhood into young adulthood, much of the work can be diminished, resulting in a range of issues continuing into adulthood [32].

In relation to the SDH, with some exceptions, across selected policies, there was little action. The areas addressed, to a limited degree, include early childhood development, education, parental workplace conditions, healthy settings, and housing. Access to health care was present but was mostly associated with acute care services. The major silences across selected policies include, but are not limited to, socio-cultural conditions such as racism, gender inequality, bullying and other forms of discrimination; and corporate, environmental and global forces including climate change, marketing to children and materialism (see Table 1) [3, 5, 10, 12]. While there is some mention of the above issues in the background section and suggested frameworks in policies, there is little to no commitment to action.

We argue, particularly for children and youth, a more balanced approach is needed. However, the long term and complex nature of adopting a SDH approach is challenging, and health departments often see some of this work to be outside the scope of their departmental responsibility [6]. Therefore, to make progress on the SDH, we need to continue to produce evidence demonstrating how policy actors can work across departments and navigate the Australian policy environment and advocate for change.

One of the key findings from this study, that demonstrates a more balanced approach, is the increased focus on housing in some states. In Australia, and across the globe, housing has become a complex policy issue, with many NGO’s calling for urgent attention to address access to safe, secure, and affordable housing [34, 35]. Recent research suggests, in Australia, over a million lower income households are paying housing costs exceeding the commonly-used affordability benchmark of 30% of household income [34]. This percentage is elevated to 40% at a global level [13]. Youth are particularly vulnerable to ‘insecure’ housing experiences [36, 37] contributing to a range of negative health outcomes [38]. This is because they are most likely to be low income earners and renting, therefore, they fall into the category of spending more than 30% of their income on housing. Children living in families that are experiencing housing insecurity, are also vulnerable. A recent publication suggests people under 35 years of age, Aboriginal and Torres Strait Islander people, and women and families fleeing family violence are more likely to experience homelessness. Furthermore, in Australia there is a national shortage of just over 400,000 homes affordable for Australians who are experiencing homelessness or living on the lowest incomes [39]. Therefore, even a hint at governments taking action on housing is welcomed. A recent case study on a pop-up shelter for youth in NSW, suggests in order to create a more resilient and sustainable housing industry we need to adopt a multi-stakeholder approach where government and other sectors work together to find innovative solutions to this problem [40]. More advocacy and research is needed in the area of housing for children, youth, and families. This issue is linked to SDG Goal 11 which aims to ‘make cities and human settlements inclusive, safe, resilient and sustainable’, and in particular target 11.1 to ‘ensure access for all to adequate, safe and affordable housing and basic services and upgrade slums’ [13]. We argue, there is currently a window of opportunity that supports the exploration of housing strategies within the Australian child and youth health policy environment, and these efforts should continue, and will result in better health outcomes for all.

Therefore, recommendations for future policy development for children and youth in Australia in both health and education departments are to: (a) invest time in understanding the current windows of opportunity for a SDH approach within the Australian health/education policy environment (b) expand work on SDH that have been recognised in current child and youth health, and selected education wellbeing policies, through advocacy efforts and future policy and program development (c) look to address those SDH not currently being addressed by building an evidence base and networks to advocate for change.

## Conclusions

Across 26 Australian child and youth health, and selected education wellbeing strategies, only 10% of strategies addressed the SDH. The SDH addressed to some degree include ECD, education, parental workplace conditions, healthy settings, and housing. There is a relatively even focus on all developmental stages, with increased attention on youth. Equity is acknowledged across most policies with some groups receiving more attention including Aboriginal and Torres Strait Islander children. Therefore, while there have been some improvements, the Australian government continues to focus predominantly on a siloed, acute care approach, demonstrating a lack of policy action on the SDH. However, child and youth voices are being recognised more during policy development, the link between health and education policy is getting stronger, and there is a window of opportunity to address housing, with a specific focus on youth. The above findings will be useful for policy makers as they formulate health, and education wellbeing policy, in the future.

## Data Availability

All data analysed during this study (ie health and education policies) were available on non-restricted government websites at the time the study was conducted. All policies analysed during the current study are available from the corresponding author on reasonable request.

## Abbreviations

SDH: Social determinants of health
HE: Health equity
SDG: Sustainable Development Goals
ECD: Early Childhood Development
CALD: Culturally and linguistically diverse
LGBTIQ+: Lesbian, Gay, Bisexual, Trans, Intersex and Queer
OECD: Organization for Economic Cooperation and Development
ACT: Australian Capital Territory
VIC: Victoria
NSW: New South Wales
WA: Western Australia
NT: Northern Territory
QLD: Queensland
SA: South Australia
TAS: Tasmania
